# The brain in social context: A systematic review of substance use and social processing from adolescence to young adulthood

**DOI:** 10.1016/j.dcn.2022.101147

**Published:** 2022-08-13

**Authors:** Sarah J. Beard, Leehyun Yoon, Joseph S. Venticinque, Nathan E. Shepherd, Amanda E. Guyer

**Affiliations:** a Center for Mind and Brain, University of California, Davis, 267 Cousteau Pl, Davis, CA 95618, USA; b Department of Human Ecology, University of California, Davis, 301 Shields Ave, Davis, CA 95616, USA

**Keywords:** Substance use, Adolescence, Brain, Reward, Social influence, Peer rejection

## Abstract

Substance use escalates between adolescence and young adulthood, and most experimentation occurs among peers. To understand underlying mechanisms, research has focused on neural response during relevant psychological processes. Functional magnetic resonance imaging (fMRI) research provides a wealth of information about brain activity when processing monetary rewards; however, most studies have used tasks devoid of social stimuli. Given that adolescent neurodevelopment is sculpted by the push-and-pull of peers and emotions, identifying neural substrates is important for intervention. We systematically reviewed 28 fMRI studies examining substance use and neural responses to stimuli including social reward, emotional faces, social influence, and social stressors. We found substance use was positively associated with social-reward activity (e.g., in the ventral striatum), and negatively with social-stress activity (e.g., in the amygdala). For emotion, findings were mixed with more use linked to heightened response (e.g., in amygdala), but also with decreased response (e.g., in insula). For social influence, evidence supported both positive (e.g., cannabis and nucleus accumbens during conformity) and negative (e.g., polydrug and ventromedial PFC during peers’ choices) relations between activity and use. Based on the literature, we offer recommendations for future research on the neural processing of social information to better identify risks for substance use.

## Highlights

• Brain activity to social stimuli is crucial for substance use, yet understudied.
• Systematic review of 27 studies on social reward, emotion, sensitivity, and stress.
• Emotional stimuli in 14 studies, social reward in 3, sensitivity 5, and stress 5.
• Greater amygdala response is both a predictor and consequence of substance use.
• Young adult substance users show more neural activity during social experiences.

## 1 Introduction and scope of review

The use of substances (e.g., alcohol, cannabis, tobacco) is a public health concern that escalates from adolescence to young adulthood (Johnston et al., 2020), with consequences for academic performance, psychosocial problems, and incarceration (King et al., 2006, Squeglia and Cservenka, 2017, Thoma et al., 2011). During this time, three-quarters of deaths are attributable to substances, such as drunk driving (Heron, 2021, Stone et al., 2012). A crucial goal for understanding the etiology of substance use is to identify factors that place certain adolescents at higher risk. Given the neurobiologically-reinforcing nature of substances, one difference is neural activity when responding to reward, and when inhibiting behavior. Functional magnetic resonance imaging (fMRI) research has detailed how monetary reward- and response-inhibition-related brain function predicts use (Forster et al., 2018, Garrison and Potenza, 2014, Moeller and Paulus, 2017). Although such processes are important, use does not occur in a vacuum, as most youth begin experimenting because someone else offers (Dishion and Owen, 2002). Understanding how the brain responds to social and emotional stimuli would advance the field by identifying novel brain-based factors contributing to substance use. Existing work has primarily focused on adults over 25, and domain-general risk-taking in adolescents, highlighting the need to focus on social processes earlier on.

Substance use during adolescence ranges from short-lived experimentation to higher-risk patterns (Gray and Squeglia, 2017). Alcohol is the most prevalent type of substance used among American adolescents, followed by cannabis, and then tobacco (Grant and Dawson, 1998, Saha et al., 2018). Substance use disorders (SUDs) during adolescence are quite common, with 16 % meeting diagnostic criteria for alcohol use disorder (AUD) and/or cannabis use disorder (CUD) (Conway et al., 2016, Margret and Ries, 2016, Merikangas et al., 2010, Swendsen and Merikangas, 2000). Some experimentation is unlikely to have long-term consequences; however, certain youth will develop SUDs (Center for Behavioral Health Statistics and Quality, 2018, Johnston et al., 2020). Thus, it is important to identify risk factors for early initiation, and heavy use (Weissman et al., 2015). Past research has highlighted one such factor as brain function underlying specific psychological processes in response to different environmental cues. Behavioral work has identified a myriad of social aspects among adolescents in relation to substance use, such as peer influence (Grevenstein et al., 2020, Prinstein and Wang, 2005, Stone et al., 2012).

Our systematic review discusses the role of brain function (via task-based fMRI) in substance use during adolescence and young adulthood, emphasizing social (e.g., peer acceptance) and emotional stimuli (e.g., negative words). We assess studies of both initiation, such as neural activity predicting future use, as well as consequences, such as past use predicting future brain responses. Following an overview of theoretical perspectives to ground understanding of reviewed findings in neurodevelopmental processes and maturational brain changes, we review studies relating substance use with neural function during social reward, emotion processing, social influence, and stressful social situations.

### 1.1 Neurobiological theories of substance use in adolescents and young adults

Adolescence involves normative but significant changes in brain structure and function. This includes shifts in the distribution and density of dopamine receptors in reward-related regions such as the nucleus accumbens (NAcc) within the ventral striatum (VS; Laviola et al., 2001), and reduction of synaptic density (i.e., synaptic pruning) in cortical regions such as the prefrontal cortex (PFC) that continues into young adulthood (although at a reduced rate; Selemon, 2013). Gray matter volume among frontal and parietal cortical areas typically peaks around 10–12 years old, and then begins to decrease from adolescence into adulthood with gray matter in the PFC decreasing the latest during that window (Giedd et al., 2015, Giedd and Denker, 2015, Sowell et al., 2010). White matter volume increases steadily into the early 20 s, strengthening connections between the PFC and other regions. These neurodevelopmental changes contribute to the adolescent increase in reward-seeking and risk-taking behaviors; for example, reward-related activation in the VS peaks in mid-adolescence (Braams et al., 2015, Silverman et al., 2015). A meta-analysis pooling data from 26 studies (>800 individuals) found that both adolescents and adults recruited overlapping brain regions including the VS, insula, and posterior cingulate cortex (PCC) in response to reward-related stimuli; however, adolescents showed greater activation in these subcortical and cortical reward-related regions (Silverman et al., 2015), supporting this developmental uptick in reward sensitivity. Such normative changes in the brain might predispose adolescents to initiate the use of substances and, for some youth, increase their risk of serious consequences in adulthood (Casey and Jones, 2010, Cousijn et al., 2018, Silvers et al., 2019, Steinberg, 2010). Certain adolescents might have atypical brain characteristics that render them more vulnerable to problematic use (Gray and Squeglia, 2017, Sherman et al., 2018). For example, hypo- or hyper-activity of the VS in response to monetary reward could be a risk factor for drug-seeking behaviors (Squeglia and Cservenka, 2017, Urošević et al., 2015, Wade et al., 2019).

Several “imbalance models” have proposed that different maturational timelines of neural systems increase adolescents’ propensity for domain-general risk-taking, including substance use (Casey et al., 2008, Shulman et al., 2016, Steinberg, 2008). Some theories indicate that the reward system develops by early adolescence (VS, dorsal striatum/DS, amygdala, ventral anterior cingulate cortex/vACC, orbitofrontal cortex/OFC, medial PFC), whereas the cognitive control system develops through the twenties (lateral PFC, dorsal ACC, anterior insula/AI, inferior frontal junction/IFJ, posterior parietal cortex/PPC) (Casey and Jones, 2010, Geier, 2013, Telzer, 2016), creating a bias for the reward system until the cognitive control system catches up. Alternatively, others have proposed that the development of socioemotional arousal follows an inverted-U shaped trajectory, whereas cognitive control plateaus in mid-adolescence (Luna and Wright, 2016). Still others hypothesize that a third brain system supporting motivated behavior originates from the interplay of two opposing social-affective circuits: approach via the VS, and avoidance via the amygdala (Ernst and Fudge, 2009). Additionally, these theories have been applied to research on how salient social contexts in adolescence, such as parents and peers, influence the neural bases of adolescent risk taking (Guassi Moreira et al., 2018, Sherman et al., 2018, Van Hoorn et al., 2018); but not adolescent substance use in particular. Thus, there is a need to assess adolescent neurobiology of substance use in social contexts, particularly those involving peers. Indirectly, these theories collectively suggest that problematic use may result from individual differences that veer from normative to extreme trajectories, such as hyper-activation of the VS to reward.

Theories situated in adulthood have focused more so on substance use than risk taking per se. For example, the Reward Deficiency Hypothesis (Blum et al., 1996) holds that hypo-activity of reward-related brain regions to monetary rewards can lead individuals to seek out alternatives (e.g., alcohol), explaining why a blunted VS response may heighten risk (Bjork et al., 2007). Second, the Impulsivity Theory (Buckholtz et al., 2010, Luijten et al., 2017) proposes that hyper-activity to rewards can predispose individuals to seek out substances. The two perspectives are not mutually exclusive, and do not exclude moderators such as sex (Heitzeg et al., 2018); for example, one study found that male adolescents showed a neural response pattern suggestive of sensation-seeking whereas female adolescents showed a pattern indicative of negative affect (Swartz et al., 2020). Third, a Social Plasticity Hypothesis (Cousijn et al., 2018) contends that neuro-social mechanisms place adolescents at risk initially, but then offer protection. Mechanisms include attunement to social environments, and neural plasticity among executive control (dorsolateral PFC, PPC, dACC, inferior frontal gyrus/IFG), salience and attention (AI, vACC, OFC, VS, DS), reinforcement learning (mPFC, hippocampus), and social cognition (temporoparietal junction/TPJ, superior temporal sulcus/STS, temporal pole, posterior ACC). This hypothesis might help explain the natural desistance from substance use throughout adulthood (Chassin et al., 2004). Overall, neurobiological models are rooted in the tension between motivational and control processes, particularly in arousing situations; however, social context tends to be under-emphasized. In this paper, we review existing empirical work on social-emotional neural correlates of substance use, highlighting the salience and relevance of social-emotional information to deepen our understanding of, and guide future research on why, some adolescents engage in substance use.

## 2 Methods for locating and reviewing studies

### 2.1 Methodology for systematic literature search

**Fig. 1:**
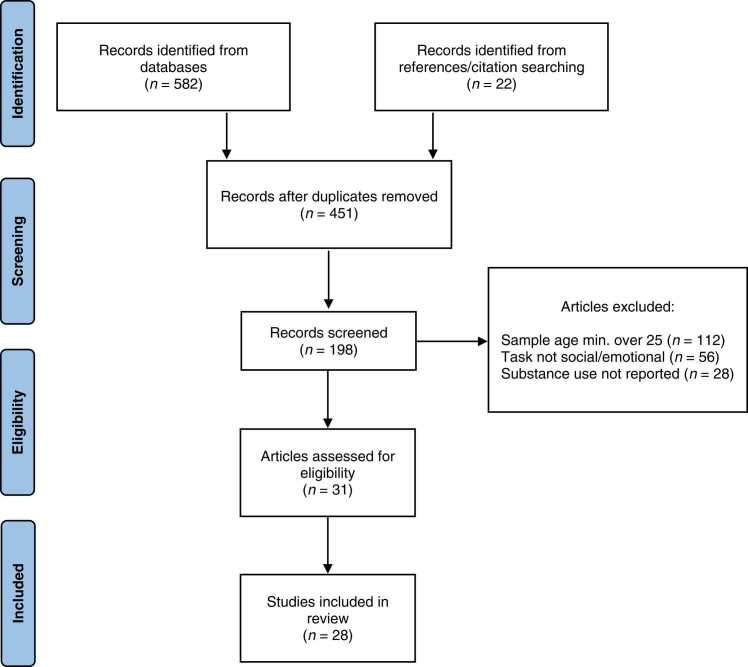
PRISMA flow diagram of study selection and inclusion. Note: For more information, visit: http://www.prisma-statement.org/.

This systematic review was performed according to guidelines from PRISMA (Preferred Reporting Items for Systematic Reviews and Meta-Analyses) (Liberati et al., 2009). The literature search was carried out using the PsycInfo and PubMed/MEDLINE databases (see Fig. 1). Initial key search terms included: substance use, drug, alcohol, cannabis, marijuana, tobacco, adolescen*, young adult, brain, neuroimaging, fMRI, social, emotion, exclusion, rejection, ostracism, and Cyberball. The final search consisted of: (((adolescen* OR "young adult" OR "emerging adult") AND (substance use OR alcohol OR tobacco OR cannabis OR cannabis)) AND (neuroimaging OR “fMRI” OR “ROI”)) AND (social task OR face* task OR social exclusion OR social feedback OR social reward) NOT ("review"[Title]). This search returned 198 entries, from which articles were excluded if the topic was not relevant to substance use (e.g., gaming disorders), if the study did not involve functional neuroimaging, and if the sample consisted entirely of adults over the age of 25. Abstracts were reviewed for discussion of substance use and neural function. If the study was relevant to the scope, the full text was retrieved and reviewed by two of the authors. Authors also reviewed articles’ references pages and “cited by” entries to locate possible articles missed in the search (*n* = 22).

### 2.2 Inclusion and exclusion criteria

Criteria included English language, empirical studies, task fMRI, at least a portion of the sample between the ages of 12 and 25, and published with a full text retrieved before August 1st, 2022. Topics include both initiation (i.e., predicting the first occurrence of cannabis, tobacco, alcohol beyond a few sips), concurrent use as correlated with neuroimaging outcomes, and the effects of substance use on neural function (i.e., predicting neural function from earlier use). This review largely does not cover studies of domain-general risk-taking (see Sherman et al., 2018), non-human animal models (see Belin et al., 2016), brain structure (see Squeglia and Cservenka, 2017), or findings in adults over the age of 25 (see Moeller and Paulus, 2017). Instead, this review builds upon previous discussions of adolescent neural function and substance use (e.g., Coronado et al., 2020; Courtney et al., 2019; Gray and Squeglia, 2017; Squeglia and Gray, 2016), contributing a new perspective about the importance of social and emotional information when understanding adolescent substance use. After applying exclusion criteria, the number of studies reviewed was 28 (see Table 2).

## 3 Results

One main goal of neuroimaging research on substance use has been to identify which patterns of neural response, and under what conditions, correlate with substance use. In doing so, a common approach has been to assess task-based responses in whole-brain analyses, as well as certain regions of interest (ROIs), particularly using tasks involving reward anticipation and receipt, and inhibition of impulsive behaviors (Tervo-Clemmens et al., 2020). The most frequently used fMRI tasks in substance use research have focused on non-social rewards; however, it is also important to examine social rewards, among other social stimuli and situations (Gilman et al., 2016). Table 1 presents the number of studies falling into different categories of stimuli/paradigm.

**Table 1:** Number of studies reviewed from the systematic literature search on social processing and substance use with samples of adolescents and young adults.

Task category	Alcohol	Cannabis	Polydrug	Total
**Social Reward**				***3***
Social Acceptance*			1	1
Pleasant Interpersonal Touch		1		1
Observing Social Interactions		1		1
**Emotional Sensitivity**				***14***
Angry and Fearful Faces	5	3	3	12
Negative Emotional Words		1		1
Negative Emotional Images	2		1	3
**Social Sensitivity**				***5***
Social Influence		1	1	2
Observing Others’ Decisions		1	1	2
Social Substance-Related Cues	1			1
**Social Stress**				***5***
Social Rejection*		1	1	2
Evaluation of Performance	2	1		2
Total	10	9	8	***28***

*Note:* Two studies assessed both social acceptance and rejection, but are counted as separate entries since the contrast of interest was different (Acceptance > Rejection versus Exclusion > Inclusion).

Table 2 presents summaries of the studies reviewed organized by social reward, emotional sensitivity, social sensitivity, and social stress, the findings from which are discussed below, respectively. Among all tasks and conditions, regions of the brain consistently implicated in substance use included the OFC, mPFC, dlPFC, dACC, AI, PPC, VS including NAcc, IFG, basal ganglia, thalamus, and hippocampus. Notably, around 55 % of studies reviewed relied on sample sizes above 60 (or 30 per group), whereas 13 % included sample sizes around 30 per group and 32 % included sample sizes smaller than 30 per group. Recent critiques have highlighted the lack of replicability when studies involve modest sample sizes with less than 30 per group (e.g., Klapwijk et al., 2021), and as such, our conclusions are mindful of methodological limitations. We expand on this issue in the Discussion.

**Table 2:** Summary of 28 studies from systematic review, with sample characteristics, measures used, neuroimaging tasks, and findings.

Authors (Year)	Sample size, age (SD), sex, ethnicity	Measure of substance use	Timing of substance use	Type of substance	Task used in fMRI	Contrast of interest	Significant brain regions	Summary of findings
**Social Reward Category**								
Jarcho et al. (2022)	*N* = 33 *M*_*age*_ = 21.88 (4.35) 64 % Female	Self-report via the Externalizing Spectrum Inventory-brief form (ESI-bf)	Concurrent substance use	Polydrug	Peer Acceptance and Rejection Task	Positive rewarding feedback > Non-rewarding feedback	VS, substantia nigra, crus cerebri	Decreased right VS response to social reward (positive rewarding feedback from a peer) was related to greater substance abuse behavior.
Zimmermann et al. (2019)	*N* = 47 (*n* = 23 cannabis) *M*_*age*_ = 23.76 (3.12) 100 % Male	Self-report	Effects of substance use on neural function	Cannabis	Interpersonal Touch Paradigm	Touch > Close Female > Male	Striatum, insula	Cannabis users showed less activity in the dorsal striatum with female experimenter touch, while non-users showed more striatal activity during interpersonal touch.
Li et al. (2021)	*N* = 460 (Human Connectome Project) *M*_*age*_ = 28.75 (3.6) 51.7 % Female	Self-report, perceived friendship	Effects of substance use on neural function	Alcohol	Social Cognition Task (shapes interacting either socially or randomly)	Social Interaction of abstract shapes > Non-Social	Right posterior insula, mOFC, left ventral precuneus	Binge drinkers, especially female, had less response in the mOFC and precuneus to social interactions of abstract shapes compared to random movement. Greater posterior insula activity was correlated with lower self-reported scores on perceived friendships.
**Emotional Sensitivity Category**								
Aloi et al. (2018)	*N* = 82 (n = 47 from a residential treatment facility) *M*_*age*_ = 16.1 (1.32) 62.2 % Male	Self-report via AUDIT, CUDIT	Concurrent substance use	Polydrug	Affective Stroop Task (Emotional Faces)	Negative Emotion > Neutral Face Positive Emotion > Neutral Face	Amygdala, precuneus, PCC, iPL	AUDIT scores were positively associated with amygdala response to emotional stimuli (both negative and positive), but negatively with dACC, dlPFC, and precuneus. CUDIT scores were positively related to activity in the PCC, precuneus, and iPL, but not amygdala.
Blair et al. (2019)	*N* = 87 *M*_*age*_ = 16.48 (1.17) 50.6 % Female	Self-report via AUDIT, CUDIT	Concurrent substance use	Polydrug	Looming Task (Emotional Faces)	Looming Stimulus > Receding Stimulus Angry Face > Neutral Face	Rostral medial frontal cortex, left fusiform gyrus, right cerebellum	CUDIT scores were negatively correlated with response to looming stimuli in the rmPFC, cerebellum, and fusiform gyrus. The pattern was similar for the amygdala, but not significant after correcting for multiple comparisons. Threatening stimuli were not related to CUDIT or AUDIT scores.
Chaplin et al. (2019)	*N* = 66 *M*_*age*_ = 12.59 (0.70) 48.5 % Female, 71.2 % White	YRBS, Teen Addiction Severity Index, urine screens, breathalyzer	Concurrent substance use	Polydrug	Emotional Photos (International Affective Picture System)	Negative Emotion > Neutral	Amygdala, ACC, AI	In girls, greater insula response to negative stimuli was associated with more substance use. Boys did not show an association between neural response to emotional stimuli and substance use.
Cohen-Gilbert et al. (2022)	*N* = 60 *M*_*age*_ = 18.9 (0.4) 55.5 % Female	AUDIT, Counseling Center Assessment of Psychological Symptoms (C- CAPS), Young Adult Alcohol Consequences Questionnaire (YAACQ)	Concurrent substance use & effects on neural function	Alcohol	Emotional Go/No-Go with emotional photos (International Affective Picture System)	Negative NoGo > Neutral NoGo	Lateral frontoparietal networks (rL- FPN; lL- FPN), dorsal attention network (DAN), salience network (SN)	Alcohol use and negative consequences of drinking were negatively associated with DAN recruitment to negative Go trials. This pattern suggests that in young adults with more problematic drinking, negative emotional information interferes more with engagement of neural networks involved in top- down attentional control, compared to those with less alcohol misuse.
Cohen-Gilbert et al. (2017)	*N* = 23 *M*_*age*_ = 18.8 (0.4) 69.6 % Female	Self-report via AUDIT, Baratt Impulsiveness Scale	Concurrent substance use & effects on neural function	Alcohol	Emotional Go/No-Go with emotional photos (International Affective Picture System)	Negative NoGo > Neutral NoGo	OFC, amygdala, MFG, frontal pole, iTG, occipital pole, precuneus, cerebellum	Binge drinking was negatively associated with activity in the dlPFC, dmPFC, and AI. This pattern emerged for negative emotion only, and not positive emotion.
Elsayed et al. (2018)	*N* = 330 (*n* = 32 early initiators) *M*_*age*_ = 13.37 (1.08) 56 % White, 44 % Other Race 44 % Girls (early initiators) 50 % Girls (late initiators)	Self-report via Substance Use Questionnaire (SUQ) and Alcohol Expectancy Questionnaire (AEQ)	Effects of neural function on substance use initiation	Polydrug	Face-Matching Task (Angry and Fearful Faces), Card-Guessing Task (Monetary Reward)	Angry and Fearful Faces > Neutral Shapes Monetary Reward > Baseline	Amygdala, VS	Adolescents who were classified as early initiators showed greater amygdala activation to fearful faces, compared to those classified as late initiators. Activity in the VS to monetary reward did not differ between groups.
Gorka et al. (2013)	*N* = 12 *M*_*age*_ = 23.2 (1.8) 83.3 % Male, 66.7 % Caucasian	In-person screening; ingestion of alcohol (0.8 g/kg; 16% volume) or placebo (dextrose with 0.0 g/kg; 1% volume ethanol as a taste mask)	Effects of substance use on neural function	Alcohol	Emotional Face Assessment Task	Fearful Faces > Neutral Faces Happy Faces > Neutral Faces	Amygdala, OFC, MTG, insula, precuneus, L supplementary motor area	Alcohol ingestion led to reduced coupling between the amygdala and right OFC to faces, regardless of emotion (both threatening and happy faces). Alcohol also led to reduced coupling between amygdala and left OFC to happy faces.
Gorka et al. (2015)	*N* = 16 *M*_*age*_ = 20.8 (2.6) 50 % Female 57.3 % Black, 31.8 % White, 6.4 % Hispanic, 5.5 % Asian	In-person screening; ingestion of THC (Marinol; 7.5 mg) or placebo (dextrose)	Effects of substance use on neural function	Cannabis	Emotional Face Assessment Task	Fearful Faces > Neutral Faces Happy Faces > Neutral Faces	Amygdala, OFC, MTG, insula, precuneus, L supplementary motor area	THC ingestion led to increased coupling between amygdala and subregions of the mPFC and rostral ACC while viewing threatening faces, compared to happy faces and neutral shapes.
Heitzeg et al. (2015)	*N* = 40 *M*_*age*_ = 20.17 (1.38) 100 % Female	Interview and self-report	Effects of substance use on neural function	Cannabis	Emotional Word task	Negative Emotion > Neutral Positive Emotion > Neutral	Insula, iPL, dlPFC, superior frontal gyrus, right calcarine fissure, frontal gyrus	Heavy cannabis users had less activation to negative words in the right insula, PFC, and occipital cortex; less activation to positive words in the right iPL; less activation of amygdala to emotion (positive or negative); and higher dlPFC to positive words. PFC to negative words mediated adolescent cannabis use and young-adult negative emotionality.
Herman et al. (2019)	*N* = 30 *M*_*age*_ = 23.40 (5.01) 70 % Female	Self-report via Alcohol Use Questionnaire	Concurrent substance use	Alcohol	Affective Stop-Signal Task (Faces), Affective Delay Discounting Task (Faces)	Fearful Faces > Neutral Faces	Lateral OFC, angular gyrus, left frontal pole, superior parietal lobule, postcentral gyrus	More binge drinking was linked to more activity during successful inhibition in the fearful context within frontal and parietal regions. More binge drinking was also related with a steeper decrease in frontal pole activity while making delayed decisions in the fearful context.
Leiker et al. (2019)	*N* = 123 *M*_*age*_ = 15.95 (1.23) 63 % Male	Self-report via AUDIT and CUDIT	Concurrent substance use	Polydrug	Face-Identifying Task (Fearful and Happy Faces)	Fearful Faces >Neutral Happy Faces > Neutral Emotional Faces (combined) > Neutral	ACC, vmPFC, lingual gyrus, medial temporal pole, iPL	Adolescents’ AUD scores were negatively correlated with vmPFC and lingual gyrus responses to emotional faces (both fearful and happy), and CUD scores negatively correlated with rostromedial PFC (including the ACC). Greater alcohol use was linked to higher iPL response to fearful faces.
Nikolova et al. (2016)	*N* = 170 *M*_*age*_ = 19.55 (1.26) 61.2 % Female 45 % Caucasian, 26 % Asian, 18 % African-American, 6 % Bi/Multi-racial, 5% other	Self-report	Effects of neural function on substance use initiation	Alcohol	Face-Matching Task (Emotion), Number-Guessing Task (Reward)	Fearful Faces > Neutral Faces Positive Reward Feedback > Negative Reward Feedback	VS, NAcc, amygdala	Higher risk for stress-related problem drinking was associated with (1) high VS response to reward and low amygdala response to threat, and (2) low VS response to reward and high amygdala response to threat.
Spechler et al. (2015)	*N* = 140 (IMAGEN) *M*_*age*_ = 14.69 (0.53) 71.4 % Male (Cannabis users), 58.6 % Male (Non-using controls)	In-person screening	Effects of substance use on neural function	Cannabis	Emotion Video facial task (video clips of faces turning angry)	Negative Emotional Stimuli > Neutral	PFC, amygdala, hippocampus, striatum	Adolescents who tried cannabis showed greater activity to angry faces in the amygdala, and those who had never tried cannabis showed lower TPJ to angry faces as well as higher activity to neutral faces in the bilateral dlPFC and right TPJ.
Spechler et al. (2020)	*N* = 1119 (IMAGEN) *M*_*age*_ = 14.41 (0.4) 54.6 % Female	Self-report via Alcohol and Other Drugs questionnaire	Effects of neural function on substance use initiation	Cannabis	Face Processing task	Angry Faces > Neutral Faces	Amygdala	Heightened amygdala response to angry faces at age 14 predicted greater use of cannabis by age 19, and the lowest amygdala activation was seen in adolescents who remained abstinent by age 19 in a dose-dependent pattern. Amygdala activation at age 19 did not differ between cannabis users and non-users.
Sullivan et al. (2022)	*N* = 66 (34 Cannabis users with 50 + lifetime occasions or 40 + in last year) *M*_*age*_ = 21.3 (2.27) 47 % Female 64 % Caucasian	Self-report via Timeline Follow Back (TLFB), Customary Drinking and Drug Use Record (CDDR)	Effects of substance use on neural function	Cannabis	Emotional Go/No-Go	Fearful Faces > Calm Faces	Rostral ACC	Decreased left and right rACC activation was found during successful inhibition with fearful faces, in cannabis users (abstinent at time of study) compared to non-users. Greater connectivity between right rACC and right cerebellum was found during successful inhibition with calm faces for male cannabis users, compared to female cannabis users.
Swartz et al. (2017)	*N* = 377 *M*_*age*_ = 19.8 (1.3) 59 % Female 48 % Caucasian, 32 % Asian, 9 % African-American, 7 % Bi/Multi-racial, 4 % other	Self-report via AUDIT	Effects of neural function on substance use initiation	Alcohol	Emotional Face Assessment Task	Fearful Faces > Neutral Faces	Basolateral and centromedial amygdala	Greater amygdala activity to fearful faces was linked to peer-reported (but not self-reported) lower extraversion and higher conscientiousness. Conscientiousness was in turn related to problem drinking in men, but not women. Amygdala response indirectly predicted males’ drinking via conscientiousness.
**Social Sensitivity Category**								
Blair et al. (2021)	*N* = 102 *M*_*age*_ = 16.54 (1.26) 66 % Male	Self-report via AUDIT, CUDIT	Concurrent substance use	Polydrug	Retaliation Task (modified Ultimatum Game)	Retaliation Phase > Baseline	vmPFC, dmPFC, AI, caudate, periaqueductal gray, middle frontal gyrus, superior temporal gyrus	AUD scores were positively associated with brain activity during retaliation in the dmPFC, AI, and caudate, representing an exaggerated retaliation response. CUD scores were not significantly associated with neural response during the retaliation task.
Chung et al. (2020)	*N* = 78 (*n* = 46 substance-naïve) *M*_*age*_ = 15.92 (0.78) 79 % White 54 % Male for substance-naïve 38% Male for substance-exposed	Self-report via YRBS	Effects of substance use on neural function	Polydrug	Gambling Task with risky and safe choices, completed alone or after viewing peers’ choices	Safe choices by peer > Solo Risky choices by peer > Solo	vmPFC, dmPFC, amygdala, temporal pole, right STS, left TPJ, precuneus, PCC	Substance-naïveté is linked to increased valuation of peers’ safer choices, as adolescents who never used substances showed stronger neural response while observing safe choices. Further, vmPFC activity while viewing peers’ safe choices was negatively related with substance exposure.
Gilman et al. (2016)	*N* = 40 *M*_*age*_ = 20.9 (2.1) 100 % Female	Self-report	Effects of substance use on neural function	Cannabis	Social Influence Task	Conformity > Deviation	NAcc, VS, caudate, PFC	Cannabis users showed more NAcc activation while following group influence, and greater activity was associated with greater cannabis use. cannabis users showed more dorsal caudate activation during the feedback phase.
Gilman et al. (2016)	*N* = 43 *M*_*age*_ = 21.1 (2.2) 52.4 % Female	Self-report, Multidimensional Iowa Suggestibility Scale (MISS)	Effects of substance use on neural function	Cannabis	Social Influence Task	Conformity > Deviation	dlPFC, dmPFC, vlPFC, vmPFC, IFG, PPC	Susceptibility to social influence was positively correlated with caudate response to social influence, and reaction time with activity in frontal and parietal regions. Cannabis users showed more IFG activity, while non-using controls showed more dlPFC activity.
Groefsema et al. (2020)	*N* = 153 *M*_*age*_ = 22.78 (1.84) 100 % Male	Online screening; Self-report	Concurrent substance use	Alcohol	Social‐Alcohol Cue‐Exposure (SACE) task, Beer‐Incentive‐Delay (BID) task	Social > Non-Social Alcohol > Soda	ACC, vmPFC, superior frontal gyrus, VS, iPL, left STS, right TPJ	Alcohol cues (versus soda) elicited more VS and vmPFC activity. Social cues (versus non-social) elicited ACC, VS, and vmPFC. Social alcohol cues elicited STS and L-IPL.
**Social Stress**								
Beard at al. (2021)	*N* = 181 *M*_*age*_ = 17.16 (0.44) 49.3 % Female, 100 % Mexican-American	Self-report	Effects of neural function on substance use initiation	Polydrug	Cyberball (Social Exclusion)	Social Exclusion > Social Inclusion	sgACC, dACC, AI	dACC activity moderated the link between anxiety and substance use, such that adolescents with lower dACC response reported greater substance use when also reporting higher anxiety.
Gilman et al. (2016)	*N* = 42 *M*_*age*_ = 21.05 (2.2) 52.4 % Female	Self- report, Multidimensional Iowa Suggestibility Scale (MISS)	Effects of substance use on neural function	Cannabis	Cyberball (Social Exclusion)	Social Exclusion > Social Inclusion	vACC, right insula	Non-cannabis using controls showed insula activity during exclusion, but cannabis users did not. Both groups had vACC response to exclusion. Conformity was positively correlated with vACC in cannabis users (but not controls).
Shakra et al. (2018)	*N* = 48 (*n* = 24 high anxiety, 24 high sensation-seeking) *M*_*age*_ = 20.4 (1.9) 48 % Female, 81 % White, 5 % Asian, 12 % Other, 2 % Unknown	Michigan Alcohol Screening Test, AUD symptoms from Structured Clinical Interview for DSM-III-R: (SCID-NP), Experimental administration of 1 ml/kg of 95% USP alcohol, p.o.	Effects of substance use on neural function	Alcohol	Face Emotion Processing Task (FEPT; Karolinska Directed Emotional Faces set), Montreal Imaging Stress Task (MIST)	Negative Faces (averaged fearful, disgusted, angry, sad) > Neutral Faces Stress > No Stress	AI, amygdala, mOFC, NAcc, perigenual ACC	Anxious young adults showed less amygdala response to threat after ingesting alcohol, which predicted problem drinking at follow-up. Anxious men (but not women) showed increased mOFC, pgACC and NAcc activity during stress. Sensation seeking men (but not women) showed decreased mOFC during stress after ingesting alcohol, which predicted problem drinking at follow-up.
Zhao et al. (2019)	*N* = 51 (*n* = 28 cannabis users) *M*_*age*_ = 25.05 (4.33) 100 % Male	In-person screening	Effects of substance use on neural function	Cannabis	Montreal Imaging Stress Task (MIST)	Stress > No Stress	Precuneus, dmPFC	Cannabis users had decreased stress-related reactivity in the precuneus, and increased connectivity between the precuneus and dmPFC. Behaviorally, they performed worse in the social stress condition, but not the no-stress condition.

*Note:* AUDIT = Alcohol Use Disorders Identification Test; CUDIT = Cannabis Use Disorders Identification Test; YRBS = Youth Risk Behavior Survey; PFC = prefrontal cortex; OFC = orbitofrontal cortex; ACC = anterior cingulate cortex; PCC = posterior cingulate cortex; PPC = posterior parietal cortex; VS = ventral striatum; NAcc = nucleus accumbens; AI = anterior insula; MTG = medial temporal gyrus; STS = superior temporal sulcus; TPJ = temporoparietal junction; iPL = inferior parietal lobule; iTG = inferior temporal gyrus; v = ventral; d = dorsal; r = rostral; l = lateral; m = medial; sg = subgenual.

**Fig. 2:**
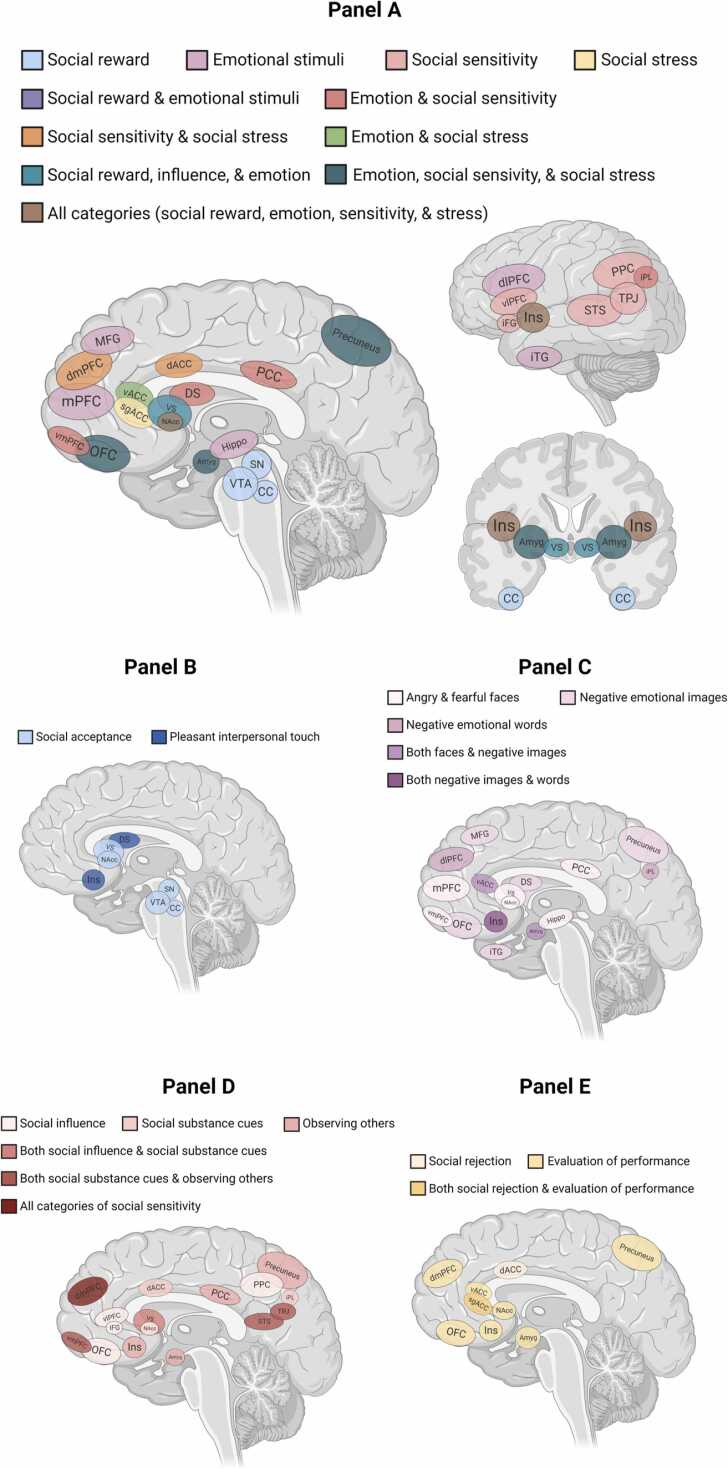
Brain regions and networks involved in substance use and processing of social and emotional information among 28 studies. Substance use during adolescence and young adulthood is associated with differences in neural activation (Panel A) to tasks involving social reward (Panel B), emotional sensitivity to faces and other charged stimuli (Panel C), social sensitivity to peer influence and perceptions (Panel D), and social stress during experiences such as social rejection and performative evaluation (Panel E). Several of these brain regions are implicated in multiple categories of stimuli, such as insula and NAcc activation emerging in at least one study across all four categories. Activity was unique to social stress among the subgenual ACC; unique to social reward among the VTA, crus cerebri, and substantia nigra; unique to emotional stimuli in the hippocampus, dlPFC, MFG, and iTG; and unique to social sensitivity in the TPJ, STS, vlPFC, PPC, and the iFG. *Note*: Amyg = Amygdala, Ins = Insula, VS = ventral striatum, DS = dorsal striatum, NAcc = nucleus accumbens, VTA = ventral tegmental area, SN = substantia nigra, CC = crus cerebri, vACC = ventral anterior cingulate cortex, dACC = dorsal anterior cingulate cortex, sgACC = subgenual anterior cingulate cortex, PCC = posterior cingulate cortex, iPL = inferior parietal lobule, STS = superior temporal sulcus, TP*J* = temporoparietal junction, OFC = orbitofrontal cortex, mPFC = medial prefrontal cortex, vmPFC= ventromedial prefrontal cortex, dmPFC= dorsomedial prefrontal cortex, lPFC = lateral prefrontal cortex, dlPFC = dorsolateral prefrontal cortex, MFG = medial frontal gyrus, iFG = inferior frontal gyrus, iTG = inferior temporal gyrus. *Note:* Created with www.BioRender.com.

See Fig. 2 for an overview of the relevant brain regions found across studies. Several of these areas overlap across multiple categories of stimuli, such as insula and nucleus accumbens activation being reported in at least one study across all four categories.

### 3.1 Neural response to social reward in relation to substance use

Social rewards have been found to engage brain regions known to respond to monetary rewards, in line with neurodevelopmental theories about maturational processes that facilitate reward sensitivity and social salience (Somerville, 2013, Nelson et al., 2016). One type of social reward particularly important in adolescent substance use is peer acceptance and social inclusion, considering how peers play a vital role in forming one’s identity and establishing romantic relationships (Fareri and Delgado, 2014, Nelson et al., 2016, Sazhin et al., 2020). The potent reward value of social acceptance has been demonstrated in cognitive neuroscience research through the use of established monetary reward tasks with social modifications, and peer acceptance/rejection or inclusion/exclusion tasks (Guyer and Jarcho, 2018). For example, similar patterns of neural activity (via EEG) to both social acceptance and monetary rewards has been reported among college students (Distefano et al., 2018) and greater striatal response (via fMRI) to peer acceptance versus rejection has been found among adolescents (Guyer et al., 2012, Guyer et al., 2014). Further, in a social variant of the Monetary Incentive Delay (MID) fMRI task (Knutson et al., 2000), greater VS activity during anticipation of social rewards moderated the link between perceived peer norms and domain-general risk behaviors, such that adolescents with high VS activity took more risks when perceiving deviant peer norms and less risks when perceiving prosocial norms (Telzer et al., 2021). In contrast, those with low VS activity took less risks, regardless of peer context. Other work using “likes” on photos (a proxy for social reward) in a task resembling the social media platform Instagram (Sherman et al., 2016, Sherman et al., 2018) revealed that adolescents display more NAcc activity in response to receiving many likes, consistent with heightened NAcc response to receiving monetary rewards. Overall, these patterns suggest that socially rewarding cues such as peer acceptance engage reward-processing brain regions in adolescents and young adults.

Two studies have investigated neural response to social rewards in relation to substance use (see Table 2). In the first study, Jarcho et al. (2022) examined neural activity to receiving both monetary reward and social reward in 33 college students. The social reward task involved viewing photographs of two peers’ faces and guessing which peer liked them, before receiving peer feedback of either a green arrow pointing upward as reward (meaning the participant chose the peer who liked them) or a white horizontal dash as non-reward (meaning the participant chose the peer who never rated them). Reduced activity in the right VS in response to experiencing a socially rewarding outcome (learning the peer liked them) was associated with greater substance use behaviors, opposite to what was found when receiving a monetary reward in which *more* activity was linked to more use. Greater striatal response to positive social feedback is normative during adolescence and young adulthood, thus it is plausible that individuals who do not recruit these neural systems during positive social encounters seek appetitive experiences through other avenues, such as substances.

The second study adapted an interpersonal touch task to assess how heterosexual men (aged 18–35) with cannabis-dependency (*n* = 23) compared to non-dependent men (*n* = 24) responded to a socially rewarding physical touch from a female experimenter (Zimmermann et al., 2019). In this paradigm, the experimenter either stood far away from the participant, close to the participant, or administered repeated soft touches to the participant’s shins. Cannabis users showed decreased dorsal striatal (DS) response to touch from the female experimenter, whereas non-users showed increased response to being touched. One interpretation is that the link between cannabis dependence and blunted striatal processing of social rewards signifies a deviation from typical striatal responding, which contributes to interpersonal deficits. Given these findings, results from studies outside our initial search criteria offer additional context for interpreting the social reward and substance use findings described above. Specifically, one study used non-social stimuli (i.e., abstract shapes) to study neural response to socially-imbued interactions in relation to substance use. Participants in the Human Connectome Project (HCP) completed a task involving watching shapes interact, either in a pattern representing social interaction, or a pattern of random movement (Li et al., 2021). Binge drinkers aged 22–36 demonstrated less response in the mOFC and precuneus to social interactions among shapes. Although this study involved abstract shapes (rather than human faces), it is worth noting that heavy drinkers showed altered engagement when viewing social interactions in this controlled setting.

### 3.2 Neural correlates of emotional sensitivity in relation to substance use

Neural activity when processing emotional cues (e.g., as depicted through facial expressions) and situations has also been examined in association with substance use, given the developmental shifts in emotionality during adolescence that coincide with brain maturation (Guyer et al., 2016), and the link between emotionality and substance use in adults (Hussong and Chassin, 1994). Effective social and emotional functioning depend on the ability to identify and be sensitive to the emotions of other people, but facial emotion recognition is impaired in adults who heavily drink alcohol and/or use cannabis (Castellano et al., 2015). Adults who heavily and frequently use substances typically struggle with maintaining interpersonal relationships, and are more sensitive to the effects of social stress (Hanlon et al., 2019, Sahani et al., 2022). There is also evidence that substance use and addiction are associated with activation of both reward-control regions *and* social-emotional regions (Courtney et al., 2019, Cousijn et al., 2018). Use of alcohol and cannabis is associated with disrupted socioemotional function, including processing of emotional faces, in adults (Miller et al., 2015) and adolescents (Leiker et al., 2019).

A larger body of literature (compared to social reward) has focused on the extent to which the brain’s response to emotion (e.g., facial expressions) predicts substance use, especially when considering stress and internalizing symptoms (Nikolova et al., 2016, Spechler et al., 2015, Spechler et al., 2019, Swartz et al., 2017). Findings suggest that relatively reduced social threat-related reactivity of the amygdala (i.e., to fearful faces) may increase risk of alcohol use or AUDs. At the same time, heightened amygdala response might buffer against VS hyper-activity and risk of stress-related problem drinking in young adults, such that impulsivity-related drinking driven by the VS is dampened down by emotional sensitivity driven by the amygdala (Gilman et al., 2008, Gorka et al., 2013, Sripada et al., 2011). Other regions of interest to be investigated from an emotional sensitivity perspective, especially given the reward literature, include the ACC, NAcc, and mOFC (Blair et al., 2019, Chaplin et al., 2019, Hardee et al., 2017, Milivojevic and Sinha, 2018, Wilcox et al., 2016).

Several studies have used a modified version of a faces-matching task (Hariri et al., 2002), which involves passive viewing of emotional faces to measure whether fear or anger elicits heightened response via the amygdala. Elsayed et al. (2018) found that adolescents who were later classified as early initiators (*n* = 21) demonstrated higher neural activity to fearful facial expressions, when contrasted with a group classified as late initiators (*n* = 231). In other words, the early versus late group had higher amygdala activity to implicit environmental signals of threat. These findings are consistent with other work showing that higher amygdala activity to threat predicted future problem drinking in response to stressful life events in college students (Nikolova et al., 2012). One study of 170 young adults examined both VS response to social-emotional reward (e.g., viewing a happy face) and amygdala response to social-emotional threat (e.g., viewing an angry face), finding one pattern in which the combined effects of high VS response to reward and low amygdala response to social threat contributed to greater risk for stress-related drinking, and a second pattern wherein low VS response to social reward and high amygdala response to social threat was also associated with stress-related drinking (Nikolova et al., 2016). Additionally, Swartz et al. (2017) found that greater amygdala activity to fearful faces was linked with higher peer-reported conscientiousness, as well as lower extraversion, in male college students. Conscientiousness in turn predicted problem drinking one year later; and amygdala response to fearful faces was indirectly associated with drinking via this pathway. Taken together, these patterns imply that heightened amygdala response to social threat may be a neural risk factor for later substance use, especially when considering stress-related motivations for use. Since facial emotion recognition is impaired in adults who heavily drink alcohol (Castellano et al., 2015), it is important to consider the role of threat-processing brain regions in the development of substance use, considering adolescents face many new stressors that may be threatening (e.g., rejection by romantic partners) and lead them to engage in substance use.

Regarding cannabis use as compared to alcohol, Leiker et al. (2019) investigated the relation between symptoms of AUD and CUD and neural processing of emotional faces in 123 adolescents in a separate task, as they identified whether a face was male or female in photos involving neutral, happy, and fearful expressions. Higher AUD scores were associated with less response to faces in the vmPFC and lingual gyrus, whereas higher CUD scores were related to less response in a region of the rostromedial PFC, which included the ACC. No differences by emotion emerged for CUD symptoms; however, AUD symptoms were positively associated with response to fearful faces in the inferior parietal lobule (iPL). Additionally, Blair et al. (2019) used a “looming task” with emotional faces (angry or neutral) that appeared to either loom toward the participant by increasing in size or recede away by decreasing in size, with the increase simulating the social threat of being approached by someone with an angry face. Adolescents’ scores on the Cannabis Use Disorders Identification Test (CUDIT) scores were positively associated with response to looming stimuli (both negative and neutral) in the rostral mPFC, left fusiform gyrus, and the cerebellum. Regardless of expression (i.e., angry versus neutral faces), neural activity was evoked when faces moved toward the participant, suggesting that someone else approaching can be perceived as threatening even if they do not display anger. Overall, use of alcohol and cannabis seems to differentially relate to deficits in neural systems involved in social stress.

While most work to date has relied upon self-reported substance use, the acute effects of substances on brain function can also be tested among adults by administering substances directly. In one experiment, 12 young adult heavy social drinkers were given either alcohol (0.8 g/kg; 16 % volume) or placebo before completing an emotional faces task (Gorka et al., 2013). Drinking alcohol led to decreased coupling between the amygdala and right OFC to emotional faces, both threatening and happy; and to decreased coupling between amygdala and left OFC to happy faces. Additionally, the same research group showed that connectivity between amygdala and OFC may mediate alcohol’s effects on social behavior. In a follow-up experiment, 16 young adults who had used cannabis at least 10 times in their lives (but were not daily/heavy users) were given either THC (7.5 mg; Marinol) or placebo before completing the faces task (Gorka et al., 2015). Consuming THC led to increased coupling of activity between the amygdala and the mPFC and rostral ACC while viewing threatening faces, compared to neutral shapes. Thus, THC might reduce threat perception and/or enhance socio-emotional regulation.

Beyond the brain’s response to emotional faces, alcohol may also acutely affect response to other types of threats, such as being evaluated by others while performing a difficult task. One study administered alcohol (1 ml/kg of 95 % alcohol) to 48 young adults who were classified either as high anxiety or high sensation-seeking, before they completed two fMRI tasks: a faces task, and the Montreal Imaging Stress Task (MIST) in which individuals performed arithmetic under time pressure, viewed a visual “performance scale,” and received negative verbal feedback from the researchers throughout the task (Shakra et al., 2017). Alcohol use was predicted by specific neural activations, such that anxious individuals showed attenuated amygdala response to angry faces, reversing their sober pattern of greater amygdala response. In the MIST, anxious individuals showed increased mOFC, pgACC and NAcc activity during social stress; and sensation-seeking men showed decreased mOFC response, which predicted problem drinking. These findings support the idea of distinct risk pathways to alcohol use, as well as sex differences given that only men showed differences by sensation-seeking. These three studies experimentally administered substances in a double-blind paradigm, providing valuable insight about short-term effects of alcohol and THC on social cognition; and more evidence that substance use both shapes, and is shaped by, neural processing of emotional stimuli.

Focusing on emotional faces and cannabis use among adolescents in the IMAGEN study, a large multi-site study as part of the Enhancing NeuroImaging Genetics through Meta-Analysis (ENIGMA) Consortium, Spechler et al. (2015) tested how cannabis-experimenting adolescents reacted to video clips of faces turning angry, compared to shapes as a control. Cannabis-experimenting adolescents demonstrated greater activity to angry faces in the amygdala than non-users, whereas non-users had higher activity to neutral faces in the bilateral dlPFC and right TPJ. For cannabis users, there was no difference in TPJ activity between angry and neutral faces, while non-users showed lower activity to angry faces. Thus, cannabis use during early adolescence is linked to hyper-reactivity to negative affect in the amygdala, and hypo-reactivity in frontal cortical regions such as the dlPFC. A follow-up study (Spechler et al., 2020) examined whether amygdala reactivity at age 14 predicted use of cannabis, alcohol, and cigarettes by age 19. Heightened amygdala response to angry faces predicted greater use of cannabis, and the lowest amygdala activation was seen in adolescents who remained abstinent in a dose-dependent pattern. This pattern was specific to cannabis use, as amygdala reactivity did not predict alcohol or cigarette use. Amygdala activation at age 19 did not differ between cannabis users and non-users, suggestive that early adolescent amygdala response might be more predictive of later cannabis use. Although adult studies have shown differences between chronic cannabis users and non-users such as decreased amygdala response (Gruber et al., 2009), developmental differences in amygdala reactivity may also exist wherein early amygdala hyper-sensitivity may predict more cannabis use but then chronic use over time dampens amygdala response. Indeed, it may be that THC and other cannabinoids have an anxiolytic role in mechanisms of fear (Phan et al., 2008), such that highly-reactive individuals seek out cannabis to regulate emotional processes.

Given the theorized role for emotionally charged experiences involving threat in driving and/or maintaining substance use (Chaplin et al., 2018, Courtney et al., 2019), some work has used emotional scenes to examine associations between neural response to threat and substance use. Aloi et al. (2018) compared neural response between 47 adolescents receiving inpatient SUD treatment and 35 adolescents from the community during an affective Stroop task, requiring participants to count the quantity of numbers on the screen next to an emotionally-charged photograph (e.g., a tarantula crawling on a man’s shoulder). Results indicated differential impacts of alcohol and cannabis abuse on the adolescent brain’s processing of threatening stimuli, such that cannabis use was positively correlated with PCC, iPL, and precuneus, whereas alcohol use was positively correlated with amygdala but negatively correlated with dACC, dlPFC, and precuneus. For adolescents who used both substances, activation to negative emotional stimuli was *negatively* correlated with alcohol use only in those with low cannabis use, whereas activity was *positively* correlated with alcohol use for those with high cannabis use. Taken together, alcohol might have a stronger impact on threat-processing regions, while cannabis might affect attention-related regions. Considering the inconsistency of these findings with the face-processing results from 140 adolescents in Spechler et al., 2015, Spechler et al., 2020, despite small but sufficient sample sizes in each study, further work is needed to disentangle how amygdala response to emotional scenes is associated with substance use over time.

In addition to faces and other visual stimuli, the link between substance use and processing negative affective information has been assessed with emotionally-charged words. Heitzeg et al. (2015) used an emotion-arousal task with negative words (e.g., danger) and positive words (e.g., hope) with 40 young adults who either never used cannabis (*n* = 20) or used it heavily, finding that users had less activation to negative words in the right insula, PFC, and occipital cortex. Prefrontal response to negative words also mediated the link between adolescent cannabis use and young adult negative emotionality. One key takeaway from these results is that heavily using cannabis as an adolescent might impair later emotional functioning, but through a cortical rather than subcortical pathway.

Finally, some research has considered the effect of emotional scenes on cognitive control, given that substances impair regulatory abilities such as inhibiting responses and pursuing goal-directed behaviors especially in emotionally-charged situations. Using a Go/No-Go task with emotionally-arousing background images, Cohen-Gilbert et al. (2017) revealed that binge drinking in 23 college students was associated with less neural response to negative images in the dlPFC, dmPFC, and ACC, suggesting an inability to effectively use regulatory regions to tune out emotional distractors. Thus, reduced activity during inhibition with emotionally-charged backgrounds might reflect a failure to regulate emotion, with implications for future self-regulation problems linked to drinking. In a separate study of 60 college freshmen, problematic drinking was linked to less neural recruitment of attention regions during negative emotion No-Go trials, and greater recruitment of frontoparietal regions (Cohen-Gilbert et al., 2022). Other work on response inhibition in 30 adults (mean age 23 years) found that greater binge drinking was associated with reduced neural response in frontal and parietal regions during inhibition against background images of fearful faces (Herman et al., 2019). Sullivan et al. (2022) also reported that cannabis-using young adults (*n* = 34) demonstrated less rostral ACC activity while successfully inhibiting against a background of fearful faces, compared to non-users (*n* = 32; mean age 21 years across both groups). Men who used cannabis also displayed more connectivity between the right rACC and right cerebellum, when the background included calm faces. Finally, another study focused on the neural correlates of the ability to delay receipt of a reward, showing that greater binge drinking was linked to a steeper decrease in frontal pole activation when making a delayed choice against a background of fearful faces; again, demonstrating the influence that emotion has on cognitive control particularly among those who engaged in extreme drinking behaviors. Overall, alcohol use impacts – and is impacted by – altered function of neural regions implicated in the top-down control of emotion.

Social relationships are rich with emotional cues of various forms and serving various functions, and adolescents draw on such cues from both their parents and peers. Information from these social contexts has been linked to the neural basis of adolescent risk taking (e.g., van Hoorn et al., 2018), which sometimes includes substance use in composite measures. With regard to substance use specifically, parental relationships are an important context particularly given the association between negative emotional experiences and substance use. For example, negative parenting behaviors such as criticism and harsh vocal tone have been found to predict longitudinal increases in substance use across adolescence (Pettit et al., 2001). Emerging work has extended understanding of this behavioral pattern by focusing on neural mechanisms. Specifically, Chaplin et al. (2019) examined how 66 young adolescents’ conversations with a parent about previous conflicts were associated with neural response to negative photos (e.g., a gun in a mouth), as well as how neural response was linked to substance use. Maternal harshness was associated with more right ACC activity in girls while viewing negative images, and less anterior insula and left ACC activity in boys. Greater activity in the insula predicted more substance use in girls, but not in boys. For girls, insular response may reflect greater internalizing of negative emotional experiences, and this high emotional arousal could lead to more substance use (i.e., coping with emotional distress). Overall, these findings suggest that the adolescent brain’s response to negative emotionally-charged images is relevant for substance use, modulated by parental behaviors such as low warmth; but with girls following an internalizing pathway, similar to studies of reward response by sex revealing different pathways (Swartz et al., 2020).

Peer relationships take on heightened salience in adolescence (Nelson et al., 2016), and represent the most common contexts in which substance use begins. One core feature of strong friendships is emotional closeness (Flores and Berenbaum, 2014), as feeling close during conversations creates more positive affect among friends. While not directly testing substance use, two studies explored how adolescents’ risky behaviors (a composite including substance use) were associated with neural response while viewing their best friend’s positive affect during a conversation about past experiences (Ambrosia et al., 2018, Flores et al., 2018). Adolescents’ (*N* = 50) neural response in the vlPFC to a friend’s positive affect was linked to higher engagement in risk-taking behaviors (Ambrosia et al., 2018); however, substance use independent of other domains of risk-taking behaviors (e.g., sexual risk-taking) was not tested directly, limiting conclusions about the role of substance use specifically. Nonetheless, the task used in these studies is a good example of how research can simulate adolescents’ real-life experiences and explore connections to their health behaviors.

### 3.3 Social sensitivity in the brain in relation to substance use

Despite substantial behavioral evidence that peers constitute the primary contextual risk factor for adolescent substance use (Chein et al., 2011, Telzer et al., 2018, Welborn et al., 2015), few neuroimaging studies have directly examined how neural processing of experiences with peers predicts substance use in adolescents. As Telzer et al. (2018) highlight, this is a critical limitation given that adolescents’ decision-making occurs when they are with their peers, family, and teachers and can be compromised in highly emotionally-charged contexts. Substance use overwhelmingly occurs when with one’s peers, such as a friend offering a sip of beer at a party. Further, susceptibility to social influence is an important risk factor for the development of substance use disorders, such as CUD (Gilman, 2017). Experimental studies have demonstrated that adolescents engage in higher levels of risky behaviors and make more impulsive decisions in the laboratory when accompanied by their friends (e.g., implicit peer influence) or receive peer feedback (e.g., explicit peer pressure to take risks) (Gardner and Steinberg, 2005, Knoll et al., 2017, Smith et al., 2015, Somerville et al., 2018, Van Hoorn et al., 2019). Deviant peer influence increases adolescent risk-taking by modulating neural processes involved in sensitivity to reward and ability to engage cognitive control (Albert et al., 2013, Chein et al., 2011); however, less work exists on positive peer influences on risk-taking and substance use (Beard and Wolff, 2020, Chung et al., 2020), as well as modulation of the brain’s activity during social cognitive processes such as being accepted by a peer. Overall, neuroimaging studies highlight that for adolescents, engaging in risky behaviors may be experienced as particularly rewarding, whereas cognitive control may be compromised, in the context of peer interactions.

Based on the risk-taking literature, substance use may be predicted by neural response to social influence (e.g., conforming to peers’ attitudes about drugs), and is a fruitful area for future research. Thus far, work has focused on young adults. Gilman et al. (2016) tested neural activity during social influence, in 40 young adults who either never used cannabis or used it moderately. The task assessed the likelihood of following group decisions, or making independent choices, in a perception task involving a judgment or whether one line was longer than another. Behaviorally, no differences emerged, as both cannabis users and non-users were more likely to follow group recommendations; however, cannabis users showed more NAcc activation while following group influence compared to opposing the group, and greater NAcc activity was associated with greater cannabis use. Neural response to non-social monetary reward was not correlated with amount of cannabis use, suggesting that these functional alterations were specific to social influence. Overall, this study has elucidated how patterns of neural sensitivity to social influence in reward-related regions, as reported in work on healthy late adolescents (Venticinque et al., 2021), vary as a function of cannabis use. Using the same social influence task, (Gilman et al., 2016) tested neural activity while 43 participants made a perceptual choice after viewing the choices of unknown peers via photographs. In both groups (cannabis use versus non-use), greater self-reported susceptibility to peers was related to greater caudate response to social influence, and slower reaction time to make a choice related to more activity in frontoparietal regions; non-users had more activity in the dlPFC, whereas cannabis users had more activity in the IFG. Cannabis users also showed slower reaction times than non-users when going against the group after viewing peers’ faces, implying more effortful processing. Taken together, young adults who use cannabis show stronger neural responses during social influence, and stronger frontal responses during decisions to go against social influence. These two studies provide evidence that cannabis use in young adulthood is linked to atypical neural response to social influence, which is distinct from monetary reward.

Substance use may also relate to neural response during aggressive behaviors, such as retaliation after experiencing an unfair situation. One study explored whether alcohol and cannabis use (on the AUDIT and CUDIT, respectively) were linked with 102 adolescents’ neural activity while making decisions to retaliate or not, in a Retaliation Task modeled after the Ultimatum Game (Blair et al., 2021). Higher alcohol use scores – but not cannabis – were related to stronger recruitment of the dmPFC, anterior insula, and caudate during retaliation, compared to other phases. Typically, these regions are active while retaliating, but adolescents who reported higher alcohol use showed stronger engagement than those with lower use. Thus, alcohol may lead to an exaggerated response while responding aggressively to unfair offers. Behavioral results also suggested that problem drinking was positively correlated with self-reported reactive aggression and irritability. It is possible that retaliation, and aggression more broadly, is one aspect of why adults who drink heavily struggle to maintain social relationships.

Other work has focused on more positive sources of social influence, such as seeing peers make safe choices as opposed to risky choices (Chung et al., 2020). Adolescents who either never used substances (*n* = 46) or did at least one time in their lives (*n* = 32) completed a gambling task with observations of peers’ decisions, both “safe” and “risky” options. While viewing peers’ risky decisions, no differences emerged between substance-naive and substance-exposed adolescents; however, during safe decisions, adolescents who had used substances displayed less activity in the vmPFC than adolescents who had never used substances. The authors highlight that neural valuation of peers’ safe choices is more strongly linked to substance exposure than valuation of risky choices, which adds an interesting dimension to the body of work about risk-taking. This pattern of valuing safe choices was found above and beyond peer presence alone, which did not reveal differences between substance-naive and substance-exposed adolescents. Indeed, peer influence from positive sources, such as prosocial peers, might buffer against substance use for adolescents who are more susceptible to social influence (Beard and Wolff, 2020, Carlo et al., 2011, Telzer, 2016). While the task itself was not social, information from this study enhances our understanding of how substance use or naivete influences social processing and risky decisions.

Substance use is predicted by stronger neural response to substance-related cues, such as a photo of beer (Courtney et al., 2016, Konova et al., 2019, Kühn and Gallinat, 2011, Nguyen-Louie et al., 2018). One interesting modification has been to separate these cues into social and non-social cues, as done in a study of 153 young men (Groefsema et al., 2020) using a modified version of a passive viewing Cue‐Exposure task with four conditions: non-social non-alcohol images, non-social alcohol, social alcohol (e.g., two people drinking beer together), and social non-alcohol (e.g., two people drinking soda). Social drinking was measured one week before the scan, in a “Bar‐Lab” designed to look like a real bar with a confederate who offered drinks. Findings revealed greater response in the VS and vmPFC in response to alcohol versus soda cues overall, and social versus non-social cues, as well as in the ACC in response to social cues (Groefsema et al., 2020). Social alcohol cues activated the bilateral STS and left IPL, uniquely from social-soda and non-social cues. Drinking behavior, however, was not related with brain reactivity to social alcohol cues, which the authors attributed to a potential ceiling effect. The researchers speculated that heightened activation to social alcohol cues in these social-cognitive areas (STS and IPL) might reflect higher motivation towards socially meaningful stimuli, but not necessarily higher potential reward. Overall, this study points to the specificity of drinking in social situations versus solitary ones, along with the importance of measuring real-world drinking behaviors beyond self-report alone.

### 3.4 Social stress in the brain in relation to substance use

Beyond reward and cognitive processes, atypical stress reactivity and deficient regulation of stress are prominent risk factors for the initiation of substance use (Chaplin et al., 2018, Zhao et al., 2019), and are considered a hallmark of addiction (Sahani et al., 2022, Sinha, 2008, Sinha, 2011). Self-report data has indicated that the regulation of negative affective states is a primary motivational drive for alcohol use (Hussong and Chassin, 1994) and cannabis use (Simons et al., 2000); and that these coping-oriented motivations are linked to an increased risk of addictive patterns of use (Schlossarek et al., 2016, Winters et al., 2021). Additionally, rat model work has revealed that social stress (e.g., social defeat), compared to non-social stress, uniquely affects dopamine activity in the mesocorticolimbic system which then affects seeking substances such as amphetamine (Burke et al., 2011). Interpersonal relationships represent a common source of social stress for adults struggling with use (Hanlon et al., 2019), making it plausible that greater sensitivity to psychosocial stress is a risk factor for adolescents to begin substance use, and for young adults to develop problematic use.

Still, relatively few studies have examined the neural correlates of social stress in particular in association with substance use, especially in adolescents. One study using the MIST tested differences in the neural response to this socially stressful context in a sample of 28 cannabis-dependent adult men and 23 non-using controls (Zhao et al., 2019). Cannabis users showed decreased stress-related reactivity in the precuneus, as well as increased connectivity between the precuneus and dmPFC. Behaviorally, cannabis users showed poorer performance on arithmetic in the psychosocial stress condition, but not the no-stress condition. In sum, lower performance in cannabis users tracked with an attenuated stress-related increase in precuneus activity, providing evidence for stress-induced cognitive performance deficits and hypo-activity of a social-cognitive brain region (precuneus) in relation to cannabis. It is unclear whether this pattern might be a consequence of cannabis use, or a preceding risk factor with origins in adolescent development. Revisiting the findings of Shakra et al. (2019), problematic drinking was predicted by neural activity during social stress using the MIST after ingesting alcohol, but with anxious young adults showing more mOFC, pgACC and NAcc activity, compared to sensation-seeking men in particular showing decreased mOFC response.

Tasks that emulate social exclusion also contribute to our understanding of neural predictors of substance use, considering that social exclusion causes psychosocial stress. Cyberball, a task commonly used in fMRI research, is a computerized ball-tossing game in which the participant is excluded from the game after a pre-determined number of ball tosses (Williams and Jarvis, 2006). Many existing fMRI studies using Cyberball have focused on domain-general risk-taking (Falk et al., 2014, Peake et al., 2013, Telzer et al., 2021) rather than on substance use specifically. Only one study has tested if neural response to social exclusion during Cyberball predicts future substance use in adolescents (Beard et al., 2021), whereas another study tested differences in neural response to exclusion between young adult cannabis users and non-users (Gilman et al., 2016). It has been hypothesized that the social rejection experienced from exclusion may diminish self-control by recruiting those same brain regions involved in inhibition, therefore increasing vulnerability to reward-based impulsive decisions and behaviors (Edalati et al., 2018). While this pattern applies to adults as well, it could be that adolescents demonstrate particularly compromised self-control after exclusion, due to neurodevelopmental changes underlying immature impulse control (Mills et al., 2016) coupled with heightened salience of peers and other social contexts (Nelson et al., 2016). Relatedly, as per the social plasticity hypothesis (Cousijn et al., 2018), perhaps social attunement also relies on prefrontal inhibitory regions. More specifically, high social attunement can be protective against substance use in later adulthood, but increase risk for use during adolescence, because teenagers are attuned to peers’ use of substances and more likely to conform to those peers. Little is known about the neural mechanisms of social attunement to peers, however; thus, future studies are needed that probe the functionality of the brain networks engaged during tasks designed to measure an individual’s level of social attunement and capacity to change behavior according to the social environment.

The existing empirical work investigating the neural correlates of social exclusion as related to substance use has been largely limited to adult samples, with exceptions from the risk-taking literature. For example, Peake et al. (2013) showed that after an acute instance of peer exclusion in Cyberball, adolescents had greater activation in social brain regions (mPFC and TPJ) while making cautious decisions in a risky driving task, and greater activation in the TPJ when making risky decisions; but only for adolescents who reported being susceptible to peers. This pattern implies that adolescents who typically avoid conforming to peers’ attitudes and behaviors might be at lower risk of substance use after being excluded. Considering that substance use is often a highly social activity, it may be that experiences of rejection motivate adolescents to take risks to regain social status, such as binge drinking to impress friends. In a sample of Mexican-American adolescents, Beard et al. (2021) tested whether neural response to social exclusion moderates the association between anxiety symptoms and increased substance use from middle to late adolescence. Adolescents with higher anxiety reported a greater relative increase in use, when also demonstrating low dACC response to exclusion, relative to high dACC response. Thus, blunted dACC response to social exclusion may serve as a neural susceptibility marker of altered conflict monitoring or emotion regulation in middle adolescence that, in combination with high levels of anxious feelings, elevates risk for onset of and/or increased substance use by late adolescence.

Regarding early adults, Chester and DeWall (2014) found that 37 college students with greater activation in the right vlPFC during social exclusion were more likely to have greater subsequent activity in the NAcc during a separate substance-cue reactivity task (i.e., while passively viewing images of alcohol and other drugs). Moreover, they reported less functional connectivity between the right vlPFC and NAcc, specifically in response to alcohol and other appetitive cues. The authors suggested that social exclusion may impair self-regulation, given that adults who exerted more regulatory effort (via greater vlPFC recruitment) were then more vulnerable to reward cues. Edalati et al. (2018) suggested that social rejection increases the tendency toward rewarding impulses, such as alcohol cravings, by routing inhibitory mechanisms toward different functions, such as inhibitory control of negative, self-reflective thoughts. Indeed, work has reported in that adult crack-cocaine users show greater activation than non-users during social exclusion in the right caudate, medial frontal gyrus, and left ventral lateral frontal gyrus (Hanlon et al., 2019).

Activation in brain regions that process social rejection and acceptance relates to substance use, both in predicting future use among adolescents and in comparing neural responses in adult substance users versus non-users. To date, the most direct test of differences in brain activity during exclusion have been conducted among young adults. Gilman et al. (2016) tested the relation between neural response to social exclusion and substance use among 42 college students who either never used cannabis or used it regularly. Cannabis users demonstrated atypical neural processing of social exclusion during Cyberball, such that non-using participants showed significant activation in the insula during exclusion compared to inclusion, whereas cannabis users did not show significant activity in the insula (Gilman et al., 2016). Both groups showed greater vACC activation; however, and notably, only the cannabis group displayed a correlation between vACC activation and self-reported peer conformity. Thus, reduced neural sensitivity in the insula to social exclusion might be indicative of cannabis users being less explicitly aware of social norms. Beyond the measure of peer conformity included in the study, a worthwhile extension might be to consider other avenues of social functioning, such as reactivity to peer influence and different social stressors such as punishment from teachers or discrimination by peers. Overall, considering the heightened salience of social rejection during adolescence, these patterns would likely be seen in younger samples, perhaps with even stronger effects. More broadly, social processes are crucial to consider, particularly for adolescents who are more attuned to peer behaviors.

To our knowledge, fMRI studies relying on the MIST and Cyberball are the only ones to investigate how substance use is associated with neural activity during experiences of social stress. While not the focus of the present review, we want to acknowledge work that has measured physiological stress sensitivity, such as functioning of the hypothalamic–pituitary–adrenal (HPA) axis as measured with cortisol. The HPA axis is a central component of the body’s neuroendocrine response to stress, and thus measuring cortisol is a way to measure sensitivity mediated by the brain (Van Leeuwen et al., 2011). Conceptually, Chaplin et al. (2018) have proposed that domain-general (i.e., non-social) stress reactivity and substance use can follow two pathways: (1) adolescents develop a heightened physiological reactivity to stress and seek out substances to cope, and (2) adolescents develop a blunted physiological reactivity to stress and seek out substances to increase arousal. Such pathways involving brain function have yet to be tested using fMRI, but some work has examined cortisol response to social stressors, using the Trier Social Stress Task (TSST; similar to the MIST) in which participants perform a task involving social-evaluative threat (e.g., giving a speech to an audience of unfamiliar adult judges). For example, adolescents who used cannabis at least once showed lower cortisol reactivity during the TSST compared to abstinent adolescents (Van Leeuwen et al., 2011). A similar pattern emerged for tobacco use in a different study of adolescents, as daily smokers showed lower cortisol reactivity (Evans et al., 2013), following a blunted-reactivity pathway. Sex differences are also key, as girls may be more likely to follow the high-reactivity pathway, whereas boys are more likely to follow the low-reactivity pathway. Such differences, however, need further exploration, especially in fMRI research.

## 4 Discussion

Adolescence involves a normative increase in risky behaviors including substance use, and young adulthood involves a steep increase in substance use. Nonetheless, considerable individual differences exist in substance use patterns and these differences likely relate to variability in neural responsivity (Figure 2 Panel A) to cues involving non-social and social rewards (Panel B), faces and other emotionally charged stimuli (Panel C), peer influence and social perceptions (Panel D), and social stress involving experiences such as social rejection (Panel E). Broadly, substance use is associated with differences in neural activation to cues involving non-social and social rewards, faces and other emotionally charged stimuli, peer influence and social perceptions, and social stress involving experiences such as social rejection. As Fig. 2 demonstrates, several of these brain regions are implicated in multiple categories of stimuli, such as insula and NAcc activation emerging in at least one study across all four categories. Activity was unique to social stress among the subgenual ACC; unique to social reward among the VTA, crus cerebri, and substantia nigra; unique to emotional stimuli in the hippocampus, dlPFC, MFG, and iTG; and unique to social sensitivity in the TPJ, STS, vlPFC, PPC, and the iFG. Otherwise, several of the same brain regions were commonly reported for different tasks and stimulus types, in relation to substance use. In the growing literature on social reward, it is unclear if neural response to social rewards follows a similar pattern to non-social reward, or if the pattern is qualitatively different. For example, college students reported greater substance abuse behaviors when they demonstrated *less* activity in the right VS during social reward, contrary to monetary reward in which more use was linked to *more* activity in the VS while receiving this reward (Jarcho et al., 2022). Regarding emotional processes, greater amygdala reactivity to fearful faces seems to be both a predictor and consequence of substance use, particularly alcohol (Elsayed et al., 2019), although less amygdala response can also predict stress-related drinking (Nikolova et al., 2016). One confound in the available research is the inability to disentangle the emotional from social component of stimuli, as common threat cues such as angry faces are also inherently social in nature.

When considering social sensitivity to processes such as peer influence, substance-using adolescents and young adults typically show stronger neural reactions to peer influence; but methodology varies widely across studies, and conclusions are difficult to draw among studies of group conformity, observations of peers’ decisions, and retaliation against unfair situations. Lastly, of the relatively few studies examining social stress, previous substance use and dependence was found to be associated with greater neural sensitivity to psychosocial stress, such that adults with heavy use typically showed more activity in brain regions processing social rejection compared to those who abstained or used only moderately (Hanlon et al., 2019, Gilman et al., 2016); but this also depended on level of peer conformity (Gilman et al., 2016). Among adolescents, substance use might follow different patterns for internalizing and externalizing problems, given that blunted dACC response combined with comorbid anxiety predicted more use (Beard et al., 2021); but with sex differences as girls (but not boys) with higher insular response reported slightly more substance use. In the reward literature, Swartz et al. (2020) found that girls’ drinking was predicted by less reward-related response, whereas boys’ drinking was predicted by more reward-related response. Given the small body of work on social processes in the brain and substance use among adolescents, more research is needed to reveal potential moderation effects. Additionally, many of the studies we reviewed relied on relatively small sample sizes with less than 30 individuals per group, which likely contributes to inconsistencies in reported findings. We discuss this point further in Future Directions.

Among adults older than 25, research on substance use and neural response to social stimuli is also sparse, but generally suggests a more consistent pattern than that found in youth. Neural activity during social exclusion appears to be higher in adults with AUD (Charlet et al., 2014; Maurage et al., 2012; Park et al., 2015), and in those who use crack-cocaine (Hanlon et al., 2019), compared to typical control participants. Further, adults who abuse alcohol displayed greater dACC and insula response to social exclusion during Cyberball (Maurage et al., 2012), as well as greater activity while viewing emotional faces in the dACC (Park et al., 2015), rostral ACC, mPFC, and precuneus (Charlet et al., 2014). Adults who use opioids showed greater activation during exclusion in the right caudate, medial frontal gyrus, and left ventral lateral frontal gyrus (Hanlon et al., 2019). Taken together, adults with substance use problems tend to show heightened brain responsivity to social stressors (Sahani et al., 2022), whereas youth show both hypo- and hyper-sensitivity to such stimuli. Conclusions are hard to draw, however, about the strength of the evidence for differences between those who use and do not use substances in their neural response to social stimuli. Although the pattern appears to be greater neural response in young adults (as well as older adults) who use substances, many of the studies reviewed relied on small sample sizes and/or cross-sectional designs, highlighting a need for replication and further evidence.

In addition to social changes (e.g., peer influence is salient during adolescence but less so during adulthood), neurodevelopmental changes likely contribute to these differences among age groups. During adulthood, the brain undergoes less change than during adolescence and young adulthood; as such, the pattern of findings related to substance use and neural function may stabilize by adulthood. Individual differences may also play a role, given that some adolescents’ brains develop on a different timeline than group averages. A second issue to consider is the effects of substance use on brain function by the time individuals progress further into adulthood. Substance use, particularly heavy and chronic use, is known to influence the structure and function of brain regions that support social decision making (Sazhin et al., 2020), such as gray matter reductions in the amygdala among adults with AUD (Wrase et al., 2008). It is possible that adolescents who are less sensitive to these social situations are more likely to start drinking heavily, which in turn impacts their brain development and leads to them being more sensitive later as adults. Next, we discuss other gaps in these bodies of work and future directions to pursue.

### 4.1 Gaps in the Existing Literature

Beyond the literature reviewed above, to the authors’ knowledge, no other studies have explored neural activation in response to social stress and social influence as predictors of substance use in adolescents. This is surprising, given the wealth of literature on the neural correlates of social influence as they relate to risk-taking in adolescents (e.g., Sherman et al., 2018), as well as evidence of self-reported social stress (e.g., experiences of interpersonal rejection) relating to substance use in adolescents (e.g., Jiang et al., 2016) and young adults (e.g., Milivojevic and Sinha, 2018). Social situations are highly salient during adolescence, and substance use rarely occurs in solitude without peers, so it is reasonable to expect that neural response to social influences would predict substance use. Additionally, although many studies have characterized the role of neural response to monetary reward, less is known about how striatal responses to social reward (e.g., peer acceptance) are associated with reward sensitivity and substance use (Distefano et al., 2018, Jarcho et al., 2022, Sazhin et al., 2020), particularly in younger adolescent samples. Lastly, as demonstrated in Table 1, we found no studies that examined tobacco use individually and not as part of a composite polydrug score. Although the relation between tobacco use and social stress has been tested via cortisol (Evans et al., 2013), it has not been tested using neuroimaging such as fMRI, which would contribute valuable information to the literature. While smoking cigarettes has declined significantly among adolescents in recent years, vaping and use of other e-cigarette products has increased (Johnston et al., 2020), and thus use of tobacco still presents a public health concern for adolescents and young adults.

In addition to variation in tasks and construct measurement, many of the studies reviewed included cross-sectional designs and had small sample sizes, which limits confidence in the reported patterns of results. Although 55 % of the reviewed studies had sufficiently large samples, including more than 30 participants per group as recommended in recent discussions of reliability and reproducibility (e.g., Klapwijk et al., 2021), 13 % of studies were near the recommended cutoff and 32 % were below with less than 30 participants per group. Thus, drawing strong conclusions from this body of evidence is constrained, and suggests that more research is needed with sufficiently powered samples. Smaller sample sizes were also concentrated in social topics, particularly social sensitivity and social stress, whereas studies with emotional stimuli often had larger samples. The ecological validity of these tasks likely impacted the ability to collect data from more individuals, particularly for younger adolescents. Bringing attention to the importance of such topics will hopefully contribute to funding opportunities and collaborations across research groups to assess the brain’s social sensitivity across the critical developmental period from adolescence to young adulthood as it relates to substance use.

### 4.2 Future directions

One promising direction toward a deeper understanding of neural function and substance use is to conduct or drawn on existing multi-site longitudinal studies with large samples; this would also address the small samples that limit existing findings. For example, the Adolescent Brain and Cognitive Development (ABCD) study in the U.S. (http://abcdstudy.org/) has 21 research sites collecting data from approximately 10,000 children beginning at ages 9–10 (Casey et al., 2018, Lisdahl et al., 2018), and has been following these participants into early adulthood, integrating structural and functional brain imaging with genetic, psychological, and physical health assessments. Neuroimaging tasks include the MID, SST, and Emotional N-Back tasks. Since this sample is largely substance-naïve (Lisdahl et al., 2018), ABCD brings an unprecedented opportunity to evaluate predictors of use onset across a diverse sample of the U.S. population, as well as how these factors influence adult outcomes (Ewing et al., 2018). Findings from the ABCD study offer a unique chance to evaluate how social experiences outside of the scanner relate to some of the fundamental neural circuits involved in adolescent substance use. In addition to its large sample size, ABCD includes participants who are ethnically and racially diverse, with baseline demographics that are similar to those of the general United States population: 50 % White, 15 % Black, 23 % Hispanic, 3 % Asian, and 9 % Mixed Race individuals. Alongside large studies like ABCD, smaller studies must continue to explore more specific socially-based task derived predictors of substance use, particularly for processes related to social stress and social reward that can be simulated using novel experimental tasks.

Neural development is a crucial context in which culturally-rooted social practices contribute to different developmental trajectories (Nketia et al., 2021, Qu and Telzer, 2017); thus, it is critical to examine how cultural values and beliefs influence neural processes related to adolescent substance use, and test if neural processes are similar or different across diverse cultural groups. Despite the importance of considering cultural values, non-White (and non-Western) individuals remain under-represented in developmental neuroimaging work (Guyer et al., 2018). Several studies we reviewed did not report sample racial/ethnicity, and of the reporting studies, only five had samples that were less than 50 % White individuals. One study of substance use and neural response to social exclusion, tested associations in a sample of 181 Mexican-American adolescents (Beard et al., 2021). Hispanic individuals constitute the largest racial or ethnic minority in the United States, and Mexican-Americans represent 64 % of the Hispanic population (Crockett et al., 2007, Martin et al., 2019, US Census Bureau, 2014). Further, Hispanic youth are at higher risk for problematic substance use (Atherton et al., 2016, Varela and Hensley-Maloney, 2009) and associated problems such as school dropout (Gonzales et al., 2004). The value of familism (*familismo*), the expectation that one's family should provide social support when needed along with the obligation to care for family (Sabogal et al., 1987), has been found to predict less alcohol use but more cannabis use among Hispanic adolescents (Soto et al., 2011, Valdivieso-Mora et al., 2016). Additionally, Schriber et al. (2018) found that familism buffered against experiencing ethnic discrimination and engaging in externalizing behaviors among Mexican-origin adolescents. In the risk-taking literature, Telzer et al. (2013) found that greater familism was related to less VS activity when receiving monetary rewards, and this reduced VS activity was related to less risk-taking. No studies to our knowledge have tested cultural values (such as familism) as related to substance use and neural function during social tasks; thus, this avenue would be fruitful for future research, along with other cultural values from non-White and non-Western populations.

Existing literature on adolescent brain function and substance use focuses mostly on non-social cognitive and motivational processes, such as neural activity during reward anticipation and response inhibition, with a relatively small body of work looking into social processes. Thus far, the literature on how the brain processes social and emotional information suggests that both hypo-reactivity and hyper-reactivity to emotional stimuli (e.g., faces) predict substance use, and individuals who use substances show atypical activation. For social processes, substance users may be more sensitive to peer influence and peer rejection; but more work is needed to disentangle unique relations with specific substances as well as trajectories of brain function and of use over time. Further, more work is needed to understand (1) how the brain’s processing of social stress relates to substance use independent of other risky behaviors, (2) how this pattern changes from adolescence to young adulthood, (3) how neural response to social reward is similar to and different from monetary reward, and (4) how susceptibility to social influence might affect the link between neural activity during social stress and substance use. Behavioral literature suggests that social stress relates to use of substances in adolescents and young adults (Clay and Parker, 2018, Giletta et al., 2017), but little is known about how the brain processes social stress in relation to substance use. Social context is crucial in the development of SUDs (Guttman et al., 2018), and developmental neuroscience offers approaches to help reveal how these complex processes begin in adolescence and continue into young adulthood.

## 5 Conclusions

Overall, substance use is a complex problem that involves individual differences in neural function embedded within the social contexts in which adolescents develop. A wealth of literature has implicated atypical functioning in reward processes, inhibition, and to a lesser extent, the processing of other people’s emotions. Social contexts, however, remain under-studied in the context of neural function and substance use. Neural response to social exclusion has been found to differ in young adults who use cannabis (Gilman et al., 2016) and older adults who use cocaine (Hanlon et al., 2019), but it cannot be determined if this is a precedent or a consequence of substance use. Beard et al. (2021) found that low dACC response to social exclusion, coupled with high self-reported anxiety, predicted greater increase in use from mid to late adolescence. The risk-taking literature has established that adolescents’ decision-making does not occur in a social vacuum, but instead under socio-emotional arousal when with peers, family, and teachers; and peers can influence neural processes involved in reward sensitivity, cognitive control, and social cognition (Chein et al., 2011, Telzer et al., 2017). Our understanding of substance use and the developing brain would benefit from more consideration of social contexts, such as tasks that measure social influence and peer affiliation. Additionally, new tasks are needed to provide greater ecological validity by building upon previous innovative tasks, such as observing responses to friends’ positive affect (Ambrosia et al., 2018, Flores et al., 2018) and viewing social cues with substances (Groefsema et al., 2020). Finally, given that journals and experts in the field recommend that neuroimaging studies include at a minimum 60 participants (or 30 per group; Klapwijk et al., 2021, Poldrack et al., 2017), larger samples are needed in studies of social context and adolescent substance use as 55 % of studies reviewed included > 60 participants. As substance use continues to be a significant public health concern with dire societal costs, a pressing goal is to identify adolescents at greater risk of problematic substance use, and determine ways to support their well-being into adulthood.

## CRediT authorship contribution statement

**Sarah Beard**: Conceptualization, Methodology, Investigation, Writing - Original Draft, Writing - Review & Editing, Visualization, Project Administration, **Leehyun Yoon**: Writing - Review & Editing, Visualization, **Joseph Venticinque**: Writing - Review & Editing, Visualization, **Nathan Shepherd**: Writing - Review & Editing, **Amanda Guyer**: Writing - Original Draft, Writing - Review & Editing.

## Declaration of Competing Interest

The authors declare that they have no known competing financial interests or personal relationships that could have appeared to influence the work reported in this paper.

## Data Availability

This review paper did not involve any data collection.
